# Introduction and benchmarking of pyMLST: open-source software for assessing bacterial clonality using core genome MLST

**DOI:** 10.1099/mgen.0.001126

**Published:** 2023-11-15

**Authors:** Adrien Biguenet, Augustin Bordy, Alban Atchon, Didier Hocquet, Benoit Valot

**Affiliations:** ^1^​ CHU de Besançon, Hygiène Hospitalière, F-25030 Besançon, France; ^2^​ Université de Franche-Comté, CNRS, Chrono-environnement, F-25000 Besançon, France; ^3^​ Bioinformatique et Big Data Au Service de La Santé, Université de Franche-Comté, F-25000 Besançon, France

**Keywords:** bacteria, cgMLST, pyMLST, software, typing

## Abstract

Core genome multilocus sequence typing (cgMLST) has gained in popularity for bacterial typing since whole-genome sequencing (WGS) has become affordable. We introduce here pyMLST, a new complete, stand-alone, free and open source pipeline for cgMLST analysis. pyMLST can create or import a core genome database. For each gene, the first allele is aligned against the bacterial genome of interest using BLAT. Incomplete genes are aligned using MAFT. All data are stored in a SQLite database. pyMLST accepts assembly genomes or raw data (with the option pyMLST-KMA) as input. To evaluate our new tool, we selected three genome collections of major bacterial pathogens (* Escherichia coli *, *Pseudomonas aeruginosa* and *Staphylococcus aureus *) and compared them with pyMLST, pyMLST-KMA, ChewBBACA, SeqSphere and the variant calling approach. We compared the sensitivity, precision and false-positive rate for each method with those of the variant calling approach. Minimal spanning trees were generated with each type of software to evaluate their interest in the context of a bacterial outbreak. We found that pyMLST-KMA is a convenient screening method to avoid assembling large bacterial collections. Our data showed that pyMLST (free, open source, available in Galaxy and pipeline ready) performed similarly to the commercial SeqSphere and performed better than ChewBBACA and pyMLST-KMA.

## Impact Statement

pyMLST software provides an open-access solution for core genome multilocus sequence typing (cgMLST). pyMLST allows users to create schemas from their own data or download existing schemas directly from https://cgMLST.org/ncs. It can also be used for multilocus sequence typing (MLST) or whole-genome MLST (wgMLST) analyses. pyMLST has the advantage of being free, open source, implemented in Galaxy and pipeline ready. The pyMLST software can also analyse reads directly using KMA software. This facilitates the analysis of assembly or non-assembly data, as required. We believe that pyMLST provides a simple and free solution for researchers or clinical biologists to investigate pathogen outbreaks or to conduct larger One Health studies.

## Data Summary

1. Code for pyMLST is available at https://github.com/bvalot/pyMLST.
2. Reads from three BioProjects downloaded from the NCBI database were used for our study: *Escherichia coli* (PRJNA795027), *Pseudomonas aeruginosa* (PRJNA644497) and *Staphylococcus aureus* (PRJNA901657).

## Introduction

The identification of epidemic clones, the search for cross-transmission and outbreak control are part of the daily work of healthcare institutions. For a long time, the reference method was pulsed-field gel electrophoresis (PFGE) [1], which can distinguish between clonal and non-clonal strains. Whole-genome sequencing (WGS), which enables gene-specific approaches for epidemiological purposes, has become more affordable. The single-nucleotide polymorphism (SNP) approach allows nucleotide-by-nucleotide identification of differences between genomes from a reference genome [2]. The core genome multilocus sequence typing (cgMLST) approach is an extension of traditional MLST. The cgMLST is more standardized and portable than PFGE [3], is more discriminant than MLST and is intended to be easier and faster to interpret than the SNP approach [4].

Several software packages are available for cgMLST analyses. ChewBBACA is a complete, stand-alone, free and open-source pipeline for gene-by-gene analysis [5]. It allows cgMLST analysis by detection of the complete coding sequence (CDS) followed by blastp analysis. SeqSphere (Ridom, Münster, Germany) and Bionumerics (bioMérieux, Sint-Martens-Latem, Belgium) are complete, stand-alone, commercial software with unavailable code. They are designed with a client/server architecture and a graphical interface. Mentalist is a free and open source pipeline that uses a k-mer approach (assembly free) to perform cgMLST [6].

We introduce here pyMLST, a new complete, stand-alone, free and open-source pipeline for cgMLST analysis with a different approach from existing software. We compared its performance with that of SeqSphere and ChewBBACA using three sets of genomes from major bacterial pathogens.

## Theory and implementation

### pyMLST software

pyMLST is standalone Python 3 software. It is developed on three-tier architecture: a database that contains the data stored on SQLite format, a library that interacts with the database and a common-line interface that uses the library. It uses tiers of software for alignment: BLAT [7], KMA [8] and MAFFT [9]. pyMLST has been released on an open-source GLP3 licence. Source code is available on github (https://github.com/bvalot/pyMLST). Packages are available on the PyPI and bioconda repositories. Wrappers have been developed for the Galaxy environment and are available on toolshed [10].

### Overview of pyMLST functionality

**Fig. 1.**
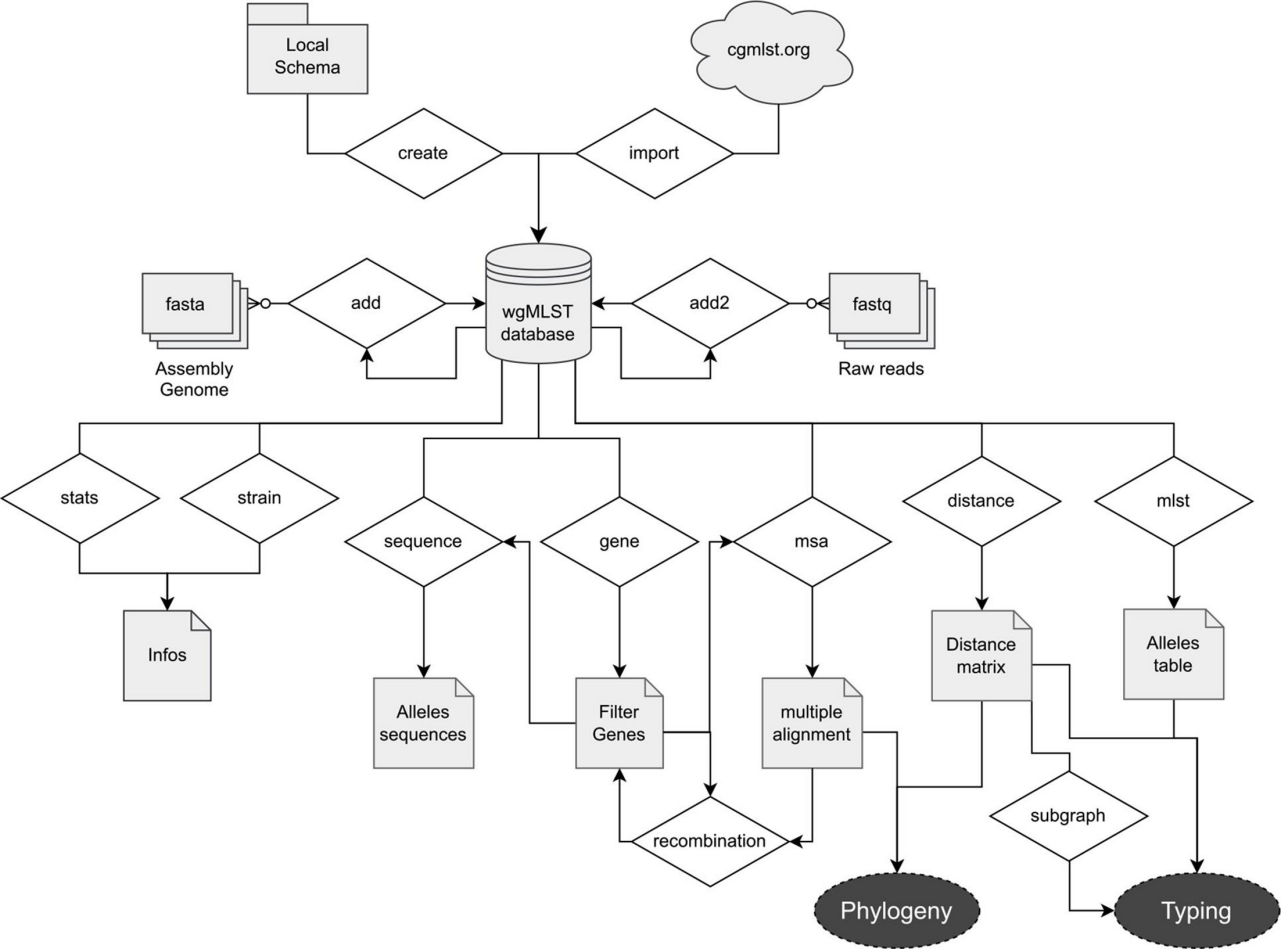
pyMLST workflow illustrating schema import/creation, adding bacterial genomes and the different analyses that can be performed.

pyMLST was developed for bacterial typing and phylogeny. Fig. 1 details the architecture of the pyMLST pipeline. Using pyMLST involves three steps: database initialization, genome import and data analysis.

#### Initialization of the cgMLST/whole-genome MLST (wgMLST) database

The database can be initiated automatically by downloading the cgMLST schema obtained from https://www.cgmlst.org/ directly into pyMLST. Alternatively, pyMLST allows the initiation of the database with a local schema obtained from published core genome analysis or by using annotated genes of a published genome close to the bacterial strains of interest.

#### Addition of strains to cgMLST/wgMLST database

pyMLST has a SQLite database allowing strain addition, modification, or removal. This allows us to iteratively increase the collection of bacterial genomes to be compared. pyMLST allows the addition of assembly strains (fasta format) using BLAT for alignment, but also of raw data (fastq format) using KMA software (hereafter called the pyMLST-KMA method). In the two cases, the first allele of each gene in the cg/wgMLST schema was used as a query to search alleles on the current strain. Partially aligned genes were realigned with MAFFT and checked (starts with a START codon, ends with a STOP codon, no frameshift). Correctly identified genes were assigned an allele number corresponding to the one in the database if the allele exists, or a new number if the allele was absent from the database.

#### Data analysis

After initiation and population of the cg/wgMLST database, pyMLST allows several analyses. First, strains can be typed and compared using allele distance matrices or MLST table. Like other tools, pyMLST calculates the distance between two strains by counting the number of genes with different alleles, ignoring missing data. Second, the sequences of selected genes (i.e. belonging to the core genome, excluding potential recombination) can be extracted for downstream phylogenetic analysis.

### Performance

pyMLST software requires few resources and could run on a personal computer with 8 GB RAM and an i5 core (e.g. on a office laptop most of the steps take a few minutes). Since strains are added to the database one at a time, this step has a linear complexity and takes 15 s per strain for a genome and 1 min for the raw reads. The advantage of database storage is that strains can be added or removed without recalculating all of the data. The most intensive computational step is distance calculation, which has a quadratic complexity and takes, for example, 30 s for 100 strains and 11 min for 500 strains.

## Method for pyMLST benchmarking

### Software

pyMLST (including pyMLST-KMA) v2.1.3 was installed following the instructions available at https://pymlst.readthedocs.io/en/latest/. ChewBBACA v3.2 was installed via conda and SeqSphere (Ridom GmbH, v8.4.2) was used in 30 day free trials. Mentalist and Bionumerics were not evaluated as the Mentalist Conda version did not run and we do not have access to a Bionumerics licence.

### Genome data

To compare pyMLST, SeqSphere and ChewBBACA, we retrieved the genome sequences from published collections of three major bacterial pathogens. A collection of 57 *Escherichia coli* ST131 isolated from animals, wastewater and humans on Reunion Island, France [11], a collection of 61 *Pseudomonas aeruginosa* ST395 isolated from patients or the environment of European hospitals [3] and a collection of 62 *Staphylococcus aureus* ST398 isolated from the nose of healthy carriers in the French community [12].

### Data preparation

Reads for the genomes from the three studies were obtained from Illumina pair-end sequencing at 150 bp and are available from the NCBI database. Reads were then trimmed with a quality threshold of 20 and adapters were removed using cutadapt v3.2.2 [13], sub-sampled to normalize the coverage of 100× (Table S1) and then assembled using SPADES v3.13.1 [14]. Contigs with coverage <2 and length <300 bp were filtered out.

### Variant calling method

To compare the pyMLST, pyMLST-KMA, SeqSphere and ChewBBACA methods, we used a variant calling method on the genes present in the pre-existing cgMLST schema available on cgmlst.org. For each collection, the reads were mapped to the following reference genomes (* E. coli* ST131 NZ_UFZF01000006.1; *P. aeruginosa* ST398 GCF_003194245.1; *S. aureus* ST398 GCA_002025125.1) using BWA-MEM v0.7.17 [15]. Then, we called variants with freebayes v1.3.4 using haploid mode [16]. SNP and insertion/deletion (INDEL) were filtered with a variant calling quality of 20 and a minimum of coverage of 5 reads. Gene alleles corresponding to cgMLST schema were then created for each sample using this polymorphism. Genes with incorrect CDSs (i.e. that included STOP codons or frameshift) or low-quality SNPs/INDELs were discarded.

### cgMLST workflows for the software

Each collection was analysed by pyMLST, SeqSphere and ChewBBACA from the same assemblies made previously. For pyMLST-KMA, we used the same sub-sampled reads as those used for the assembly. For each software package, we recovered the sequences of the alleles of the core genome of each strain. We prepared the database using cgmlst.org data for the three species. For pyMLST and SeqSphere, the database was imported automatically using dedicated options. For ChewBACCA, we downloaded the complete allele data from cgmlst.org and created the database using the PrepExternalSchema function. Analyses were performed using default parameters for each software. ChewBACCA was run as recommended with the same prodigal profile by species.

### Gene detection and evaluation of performance

For each species, the number of genes detected was counted and compared between pyMLST, pyMLST-KMA, SeqSphere and ChewBBACA. Venn diagrams of *E. coli *, *P. aeruginosa* and *S. aureus* were generated using the ggvenn R package to assess overlap between the gene detection. We compared pyMLST, pyMLST-KMA, SeqSphere and ChewBBACA with the variant calling method for each collection. We compared (i) the sensitivity, defined as the percentage of genes detected relative to the genes found by the variant calling; (ii) the percentage of false positives, defined as the percentage of genes detected that were not detected by the variant calling; (iii) the precision, defined as the percentage of genes with a same allele as found by variant calling. All of these analyses and figures were generated using the dplyr and ggpubr R packages.

### Creation of minimum spanning trees

For each collection and software pair, we kept the genes of the core genome present in >95 % of the genomes of the collection. We then created a distance matrix by counting the number of different alleles between two genomes. As usual, we assumed that if a gene was missing, there would be no allelic difference. The distance matrices were generated in R using the packages ape, stringr and dplyr. Minimum spanning trees were generated from the previously obtained distance matrices using networkx. A threshold of 10 alleles for *E. coli *, 15 alleles for *P. aeruginosa* and 6 alleles for *S. aureus* was used for clustering [17]. Cluster composition was determined from those obtained by variant calling and then compared with pyMLST, pyMLT-KMA, SeqSphere and ChewBBACA.

### Genetic distances between strains according to the software

We measured the difference of the observed distances between the variant calling and the four evaluated methods (defined as the difference in distances between the variant calling and the evaluated method for the same genome pair). Only pairs of genomes with mean distances (defined as the mean of the distance between the variant calling and the evaluated method for a genome pair) less than or equal to three times the previously defined threshold for clonality were included in this analysis. Our hypothesis is that when two strains are genetically very distant, the presence of a difference between the evaluated methods will not affect the clonal or non-clonal classification of these strains.

### Statistical analysis

For all analyses, *t*-tests were used as appropriate and a *P*-value <0.01 was considered statistically significant. No corrections were made by adjusting the *P*-value. Analyses were performed with R studio.

## Results

To evaluate the performance of our new pyMLST and pyMLST-KMA methods, we used three datasets of real data instead of simulated data from *E. coli* ST131*, P. aeruginosa* ST395 and *S. aureus* ST398. The same data were used to compare results obtained from pyMLST methods to those obtained with SeqSphere and ChewBBACA software.

### Gene detection

**Fig. 2.**
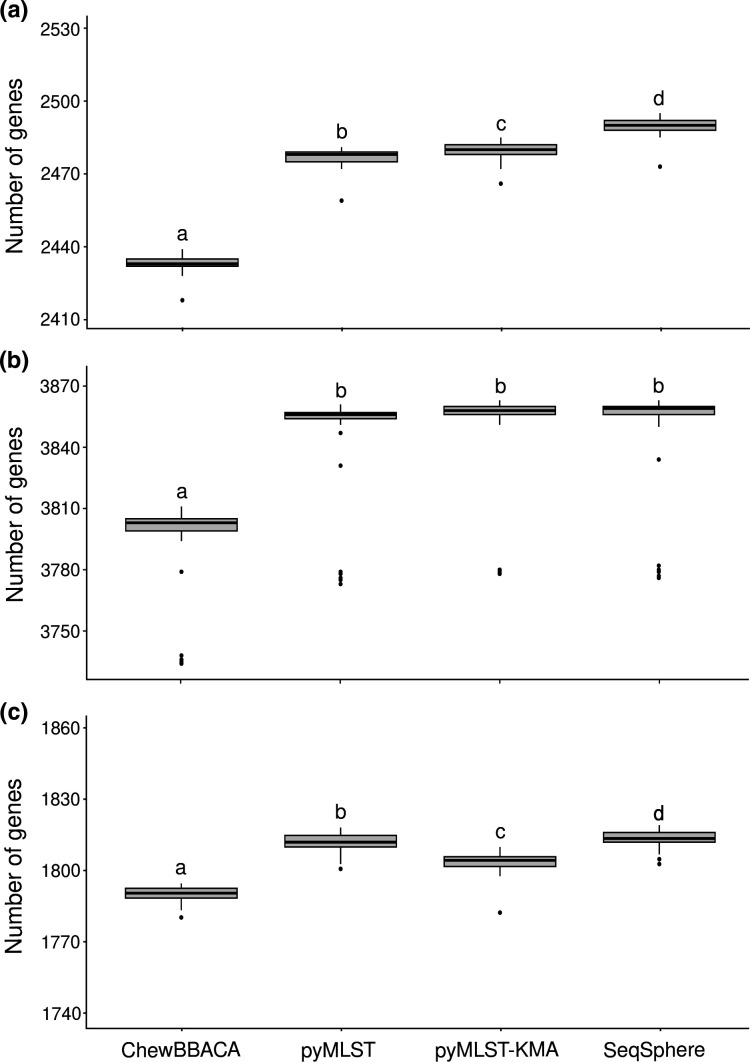
Number of genes of the core genome detected for each genome by ChewBBACA, pyMLST, pyMLST-KMA and SeqSphere for (**a**) *Escherichia coli* ST131, (**b**) *Pseudomonas aeruginosa* ST395 and (**c**) *Staphylococcus aureus* ST398. Significant differences (*P*<0.01) between two software packages are indicated by two different letters.

We compared the number of genes from the core genome detected for each strain between pyMLST, pyMLST-KMA, SeqSphere and ChewBBACA (Fig. 2). SeqSphere detected more genes than pyMLST for *E. coli* (*P*<0.01) and *S. aureus* (*P*<0.01), but we found no difference in the number of genes detected for *P. aeruginosa* (*P*=0.531). Both SeqSphere and pyMLST detected more genes than pyMLST-KMA or ChewBBACA for *S. aureus* (*P*<0.01) and *E. coli* (*P*<0.01). ChewBBACA detected fewer genes than the other methods for the three species (*P*<0.01).

Three Venn diagrams were created to compare genes detected by pyMLST, pyMLST-KMA, SeqSphere and ChewBBACA (Figs S1–S3, available in the online version of this article). A total of 2511, 3867 and 1845 different genes were detected for *E. coli *, *P. aeruginosa* and *S. aureus *, respectively, with variations according to the software (Table S2). For the *E. coli* population, most genes (2416, 96.2 %) were detected by all 4 methods, while 63 genes (2.5 %) were detected by all methods except ChewBBACA. For the *P. aeruginosa* population, 3816 genes (98.7 %) were detected by all 4 methods, while 46 genes (1.2 %) were detected by all methods except ChewBBACA. For the *S. aureus* population, 1770 genes (95.9 %) were detected by all 4 methods, 46 genes (2.5 %) were detected by all methods except ChewBBACA and 20 genes (1.1 %) were only detected by ChewBBACA.

### Evaluation of performance

**Fig. 3.**
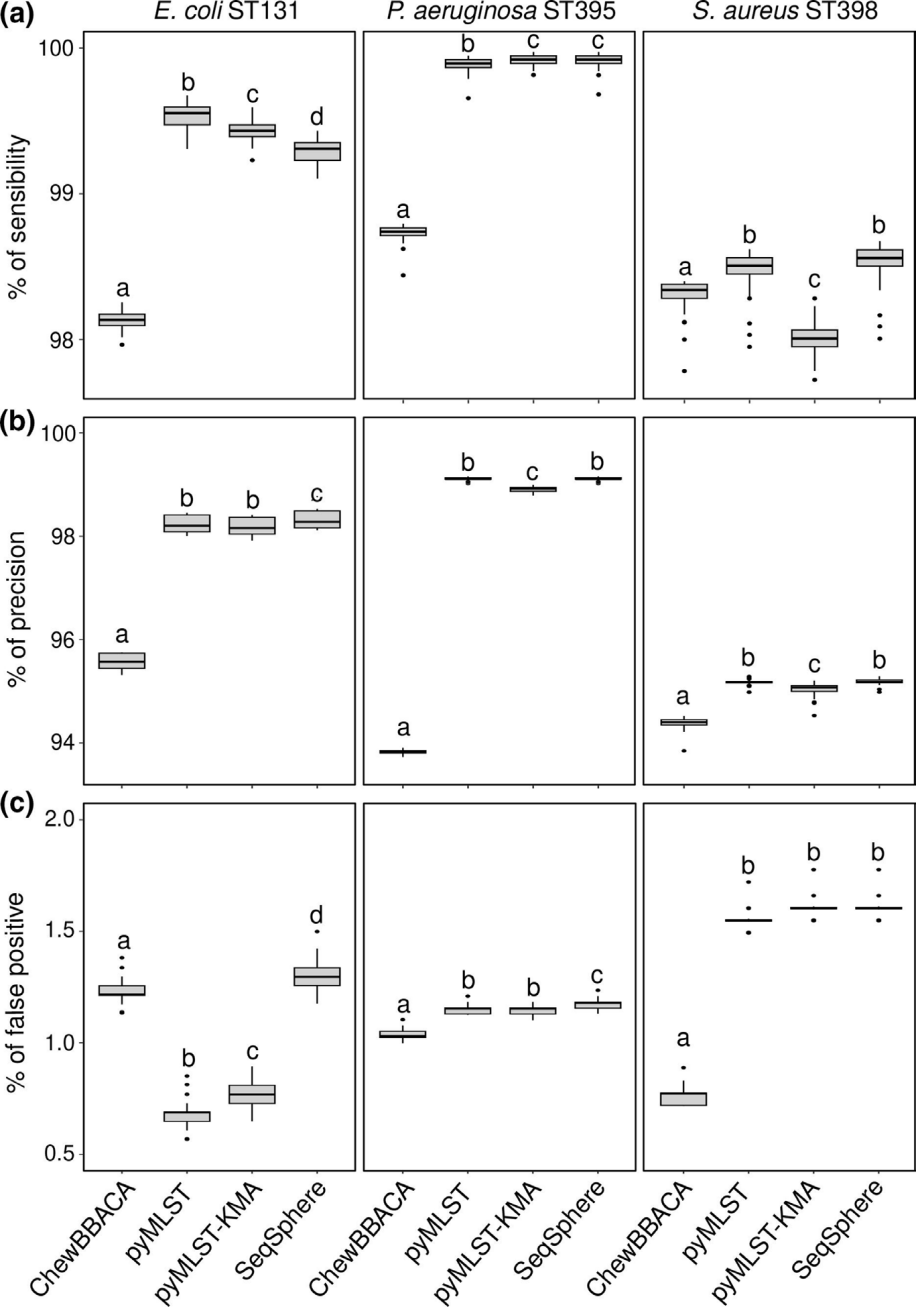
Comparison of detection of core genome genes for ChewBBACA, pyMLST, pyMLST-KMA and SeqSphere as a function of variant calling. (**a**) Evaluation of the sensitivity of the four tested methods for the detection of genes in the core genome compared to the variant calling method. (**b**) Evaluation of precision, defined as the percentage of genes with the same allele that were found between the evaluated methods and the variant calling method. (**c**) Evaluation of the false-positive rate, defined as the percentage of genes detected by the evaluated methods that were not detected by variant calling. Significant differences (*P*<0.01) between two software packages are indicated by two different letters.

To evaluated the performance of pyMLST software in comparison to other tools, we used an orthogonal approach by variant calling. Three parameters were computed: sensitivity, false discovery rates for gene detection and precision of the allele calling (Fig. 3). The sensitivity of the evaluated methods was very close between pyMLST, pyMLST-KMA and SeqSphere for the three species, even if the sensitivity of pyMLST was greater than that of SeqSphere for *E. coli* (*P*<0.01). The sensitivity of ChewBBACA was lower than that of the three other methods for *E. coli* (*P*<0.01) and *P. aeruginosa* (*P*<0.01). The percentage of false-positive gene detections was higher with ChewBBACA and SeqSphere than for pyMLST and pyMLST-KMA for *E. coli* (*P*<0.01). ChewBBACA performed better than the other three methods for false-positive rates for *P. aeruginosa* (*P*<0.01) and *S. aureus* (*P*<0.01). ChewBBACA was less precise (detection of the correct allele) than the three other methods for *E. coli* (*P*<0.01), *P. aeruginosa* (*P*<0.01) and *S. aureus* (*P*<0.01).

### Clonal detection

To test the ability of pyMLST and other tools to identify potential clonal strains, we performed MST and clustering using a cut-off distance of 10 for *E. coli *, 15 for *P. aeruginosa* and 6 for *S. aureus *.

*E. coli* MST from pyMLST and SeqSphere were very similar with 9 clusters, 24 clustered strains and very close cgMLST distances between strains in clusters (Table 1). The composition of the clusters and the distance matrix is shown in Fig. S4. Cluster 7, a cluster of two strains, was detected by SeqSphere and pyMLST with a distance of 10 for both, but was not detected by ChewBBACA or the variant calling method (genetic distances of 12 and 14, respectively, for cluster 7). PyMLST-KMA detected fewer clusters than the other methods.

**Table 1.** Number of clusters and strains belonging to a cluster detected by the variant calling, pyMLST, pyMLST-KMA, SeqSphere and ChewBBACA. The threshold for cluster detection was 10 for *E. coli *, 15 for *P. aeruginosa* and 6 for *S. aureus
*

	* Escherichia coli *		* Pseudomonas aeruginosa *		* Staphylococcus aureus *	
**Method**	**Clusters (*n*)**	**Genomes in cluster (*n*)**	**Clusters (*n*)**	**Genomes in cluster (*n*)**	**Clusters (*n*)**	**Genomes in cluster (*n*)**
**Variant calling**	8	22	9	51	0	0
**ChewBBACA**	8	22	9	50	0	0
**pyMLST**	9	24	9	51	0	0
**pyMLST -KMA**	7	18	10	48	0	0
**SeqSphere**	9	24	9	51	0	0

**Fig. 4.**
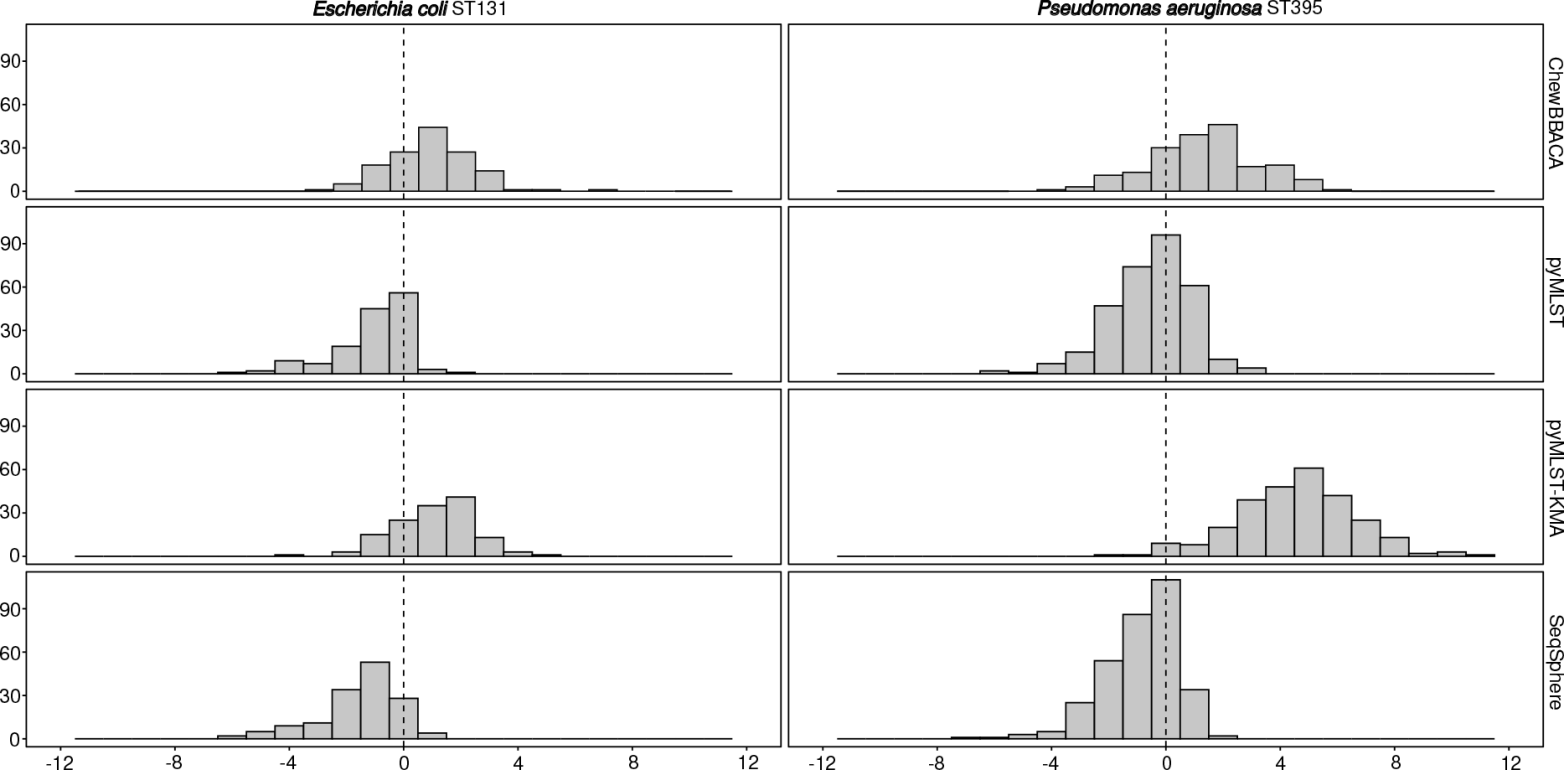
Distribution of the differences in distances per genome pair observed between the ChewBBACA, pyMLST, pyMLST-KMA, SeqSphere and variant calling methods. Only the genome pairs with an average distance ≤3 times the defined threshold for clonality (10 for *E. coli* and 15 for *P. aeruginosa *) between the evaluated methods and the variant calling method were retained.

*P. aeruginosa* MST from pyMLST, SeqSphere, and variant calling were very similar, with the same number of clusters detected (Table 1). The genetic distance between strains was higher with ChewBBACA (Fig. 4), explaining why a strain was not included in cluster 1 (Fig. S5). pyMLST-KMA created a new cluster by splitting cluster 1 into two distinct clusters.

*S. aureus* MST did not reveal any clonal strains in our ST398 population (no pair of genomes with a difference of <6 alleles).

Genetic distances between pairs of genomes observed with pyMLST were smaller compared to those observed with SeqSphere for *E. coli* (*P*<0.01) and *P. aeruginosa* (*P*<0.01) (Fig. 4). Both ChewBBACA and pyMLST-KMA showed higher genetic distances than pyMLST (*P*<0.01) or SeqSphere (*P*<0.01) for *E. coli* and *P. aeruginosa *.

## Discussion

Here we compared two variations of a new open-source software program, pyMLST, with the main software used for clonal strain identification. Using three genome collections of major bacterial pathogens, we found that pyMLST detects core genome genes in *E. coli* and *P. aeruginosa* with better sensitivity and accuracy than ChewBBACA (free open-source software). We also found that pyMLST software is as efficient as SeqSphere, a user-friendly commercial software program whose source code is not available and whose functioning is hidden. pyMLST software has the advantage of being completely free, open source, available in Galaxy and pipeline ready. SeqSphere, on the other hand, has a client/server interface, making it easy to use for phylogenetic analysis with minimal bioinformatics skills.

The differences observed with ChewBBACA results are probably due to the way it searches for genes in the core genome and assigns alleles to them [5]. Compared to pyMLST, which performs a direct BLAT for gene detection, the lower sensitivity and accuracy of ChewBBACA (Fig. 2) are probably due to its CDS detection approach. The lower accuracy of ChewBBACA for our *E. coli* and *P. aeruginosa* populations likely relies on the variation observed in the differences in distances per genome pair (Fig. 4). However, despite the apparent differences in analytical performance, ChewBBACA detects clusters of clonal strains that are almost identical to the variant calling, pyMLST and SeqSphere in our *E. coli* and *P. aeruginosa* populations.

Our pyMLST-KMA method had similar sensitivity, false positive detection and accuracy to pyMLST and SeqSphere. However, pyMLST-KMA created fewer clusters for *E. coli* and more clusters for *P. aeruginosa* (Table 1), with higher distance differences for both (Fig. 4), especially for *P. aeruginosa *. This shift in the distribution of distance differences for *P. aeruginosa* is probably due to the detection of genes that were not detected by the other methods. We do not recommend pyMLST-KMA for outbreak investigation when assembled data are available. Nonetheless, pyMLST-KMA could still be useful for data whose coverage (<30×) does not allow correct assembly or descrambling to avoid the assembly of a large number of strains by raising the clonality thresholds.

Our *S. aureus* collection had lower sensitivity, lower precision and lower false-positive rates than those observed for *E. coli* or *P. aeruginosa* analysis. These differences could be due to (i) poorer ability of the software to detect or align core genome genes, (ii) poorer selection of reference core genome genes used to initialize the cgMLST database, or (iii) a limitation of our variant calling approach for *S. aureus *. Despite these differences, the *S. aureus* population studied showed less inter-software heterogeneity than our *E. coli* and *P. aeruginosa* populations. These results highlight the need to test multiple bacterial collections to evaluate typing methods, given the variability observed.

Several parameters, such as the DNA extraction method, sequencing method, depth of coverage or quality of assembly, can influence the results of cgMLST analysis [18]. Within each collection, all genomes were extracted using the same kit and sequenced in the same Illumina run. To minimize the deviation, we chose to subsample the reads and to use the same standardized assembly across all genomes for each software program (the SeqSphere assembly method was not used). In our opinion, this approach allows us to compare the sensitivity and accuracy of the detection of the core genome between these methods.

In the absence of a gold standard for determining the clonality of clinical bacterial strains, we used an orthogonal variant calling approach. Hence, the variant calling approach ensures a completely different approach than the comparators (pyMLST, pyMLST-KMA, ChewBBACA and SeqSphere). We could have used computer-generated data or successive replications [18] to make these comparisons without variant calling. To address the need of clinical microbiologists to identify outbreaks of bacterial pathogens, we selected bacterial species of major clinical interest [19, 20] that are commonly responsible for outbreaks. However, cgMLST analysis can also be performed with pyMLST for less well-characterized bacterial species. An example is the analysis of *Proteus mirabilis* [21].

In conclusion, all the methods evaluated showed satisfactory results, with sensitivity >95 %, precision >90 % and false positive rate <2 %. With more details, pyMLST showed similar performance to SeqSphere, making it an interesting open source alternative. ChewBBACA and pyMLST-KMA performed slightly less well for the collections examined.

## Supplementary Data

Supplementary material 1
