# Supplementary material for: Introduction and benchmarking of pyMLST: open-source software for assessing bacterial clonality using core genome MLST
Source: Microb Genom. 2023 Nov 15;9(11):001126. doi: 10.1099/mgen.0.001126 (PMC10711306; doi:10.1099/mgen.0.001126)
Supplement: Supplementary material 1 [file mgen-9-1126-s001.pdf]

Table S1. Minimum, mean and maximum coverage of each genome before subsampling.

	Escherichia coli	*Pseudomonas aeruginosa*	*Staphylococcus aureus*
Min	97.0	144.0	137.0
Mean	112.4	277.6	334.4
Max	180.0	569.0	719.0

Table S2. Number of genes detected from the core genome before and after gene filtering to retain only those genes present in 95% of the population studied for a given method.

No of core genome genes detected	Variant calling	pyMLST	pyMLST-KMA	SeqSphere	ChewBBACA
Escherichia coli					
before filtration	2477	2485	2493	2498	2448
after filtration	2437	2463	2462	2470	2415
Pseudomonas aeruginosa					
before filtration	3821	3863	3865	3866	3821
after filtration	3667	3752	3761	3752	3694
Staphylococcus aureus					
before filtration	1822	1820	1823	1822	1799
after filtration	1801	1811	1797	1813	1791

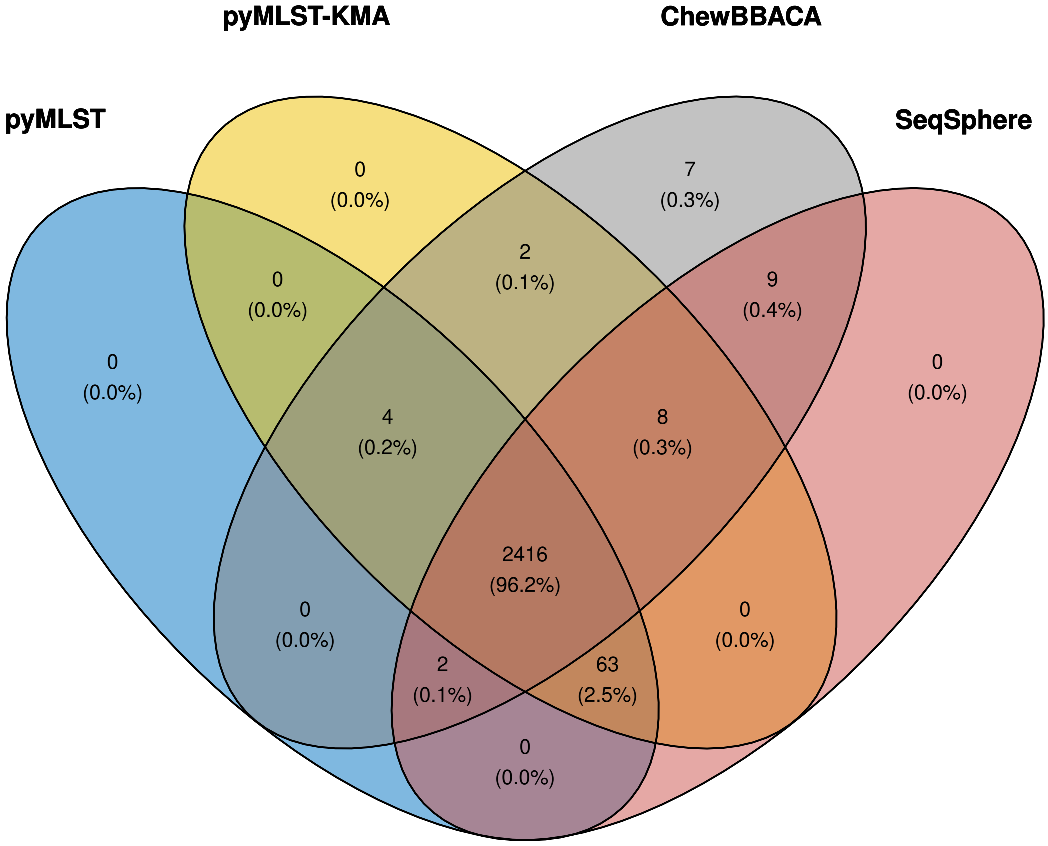

Fig. S1: Venn diagram comparing the genes used by pyMLST, SeqSphere, ChewBBACA and pyMLST-KMA for 57 *Escherichia coli*.

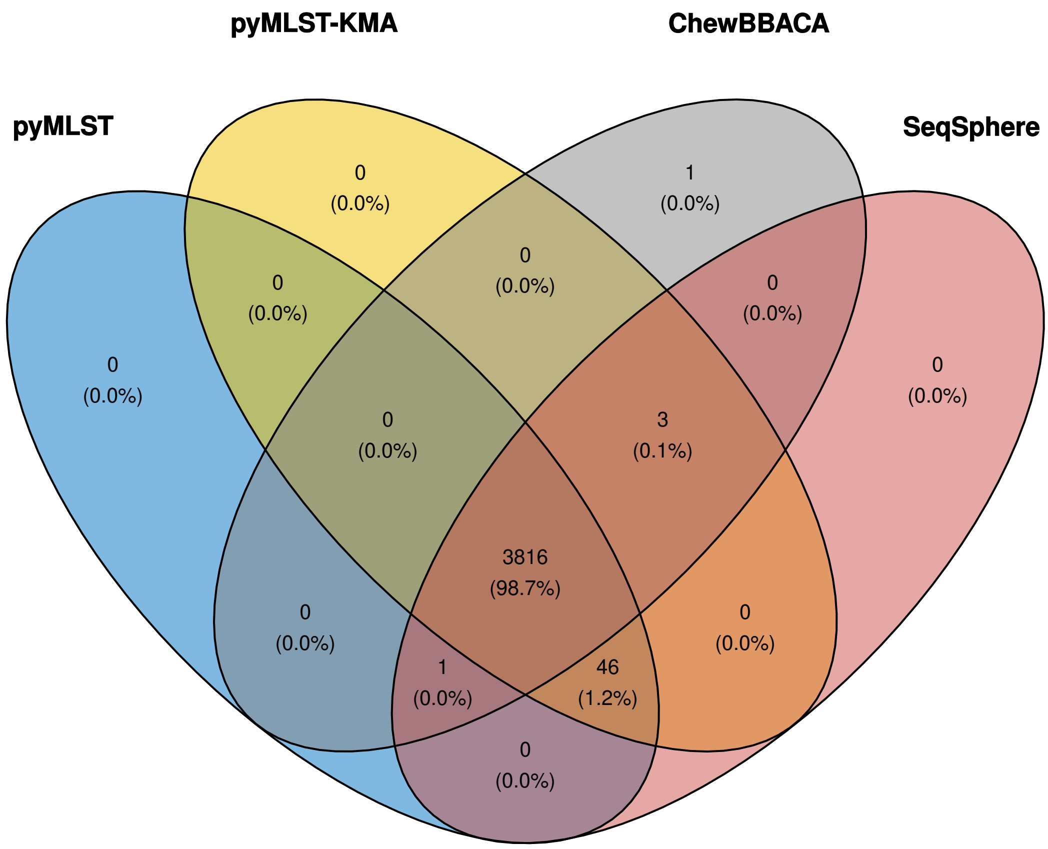

Fig. S2: Venn diagram comparing the genes used by pyMLST, SeqSphere, ChewBBACA and pyMLST-KMA for 61 *Pseudomonas aeruginosa*.

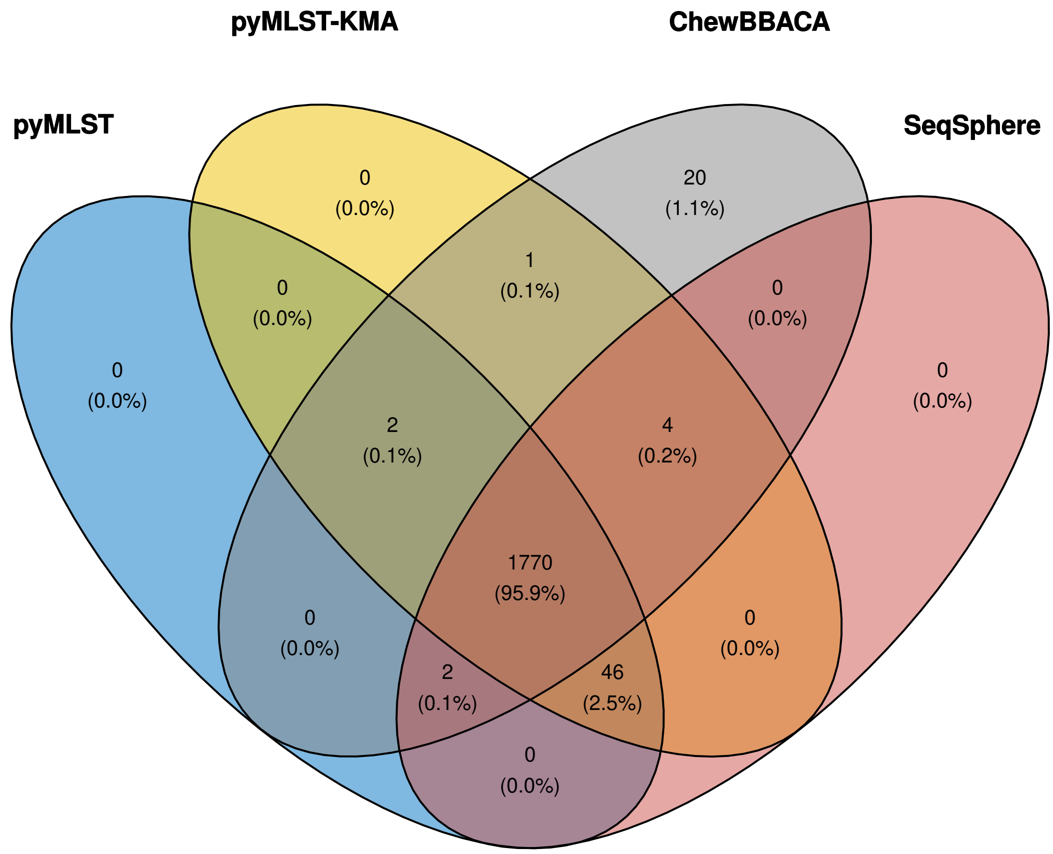

Fig. S3: Venn diagram comparing the genes used by pyMLST, SeqSphere, ChewBBACA and pyMLST-KMA for 62 *Staphylococcus aureus*.

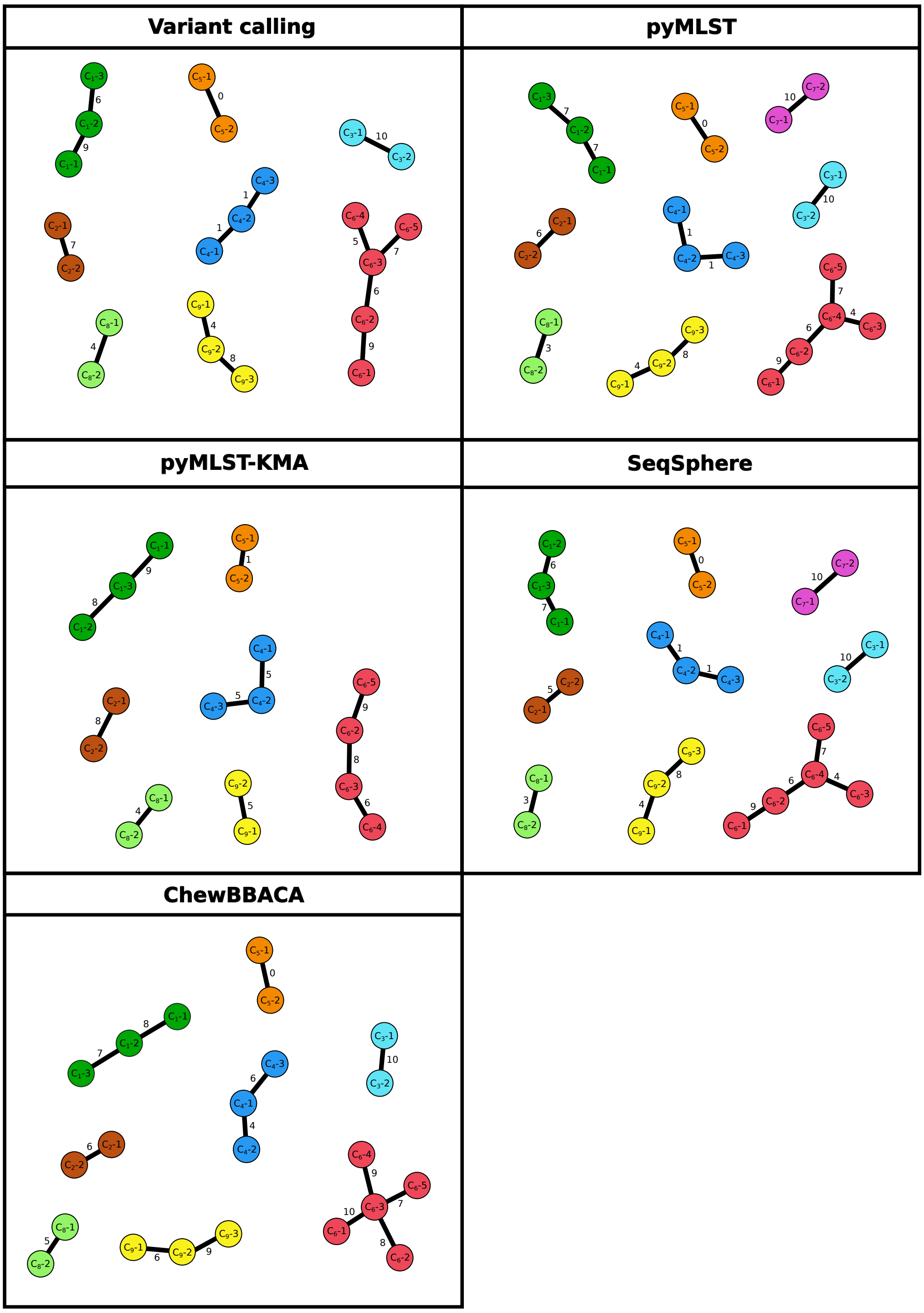

Fig. S4: Minimum spanning trees of 57 Escherichia coli core genomes using pyMLST, SeqSphere, ChewBBACA, pyMLST-KMA and the variant calling approach. Only genes present in more than 95% of the genomes in our collection were retained.

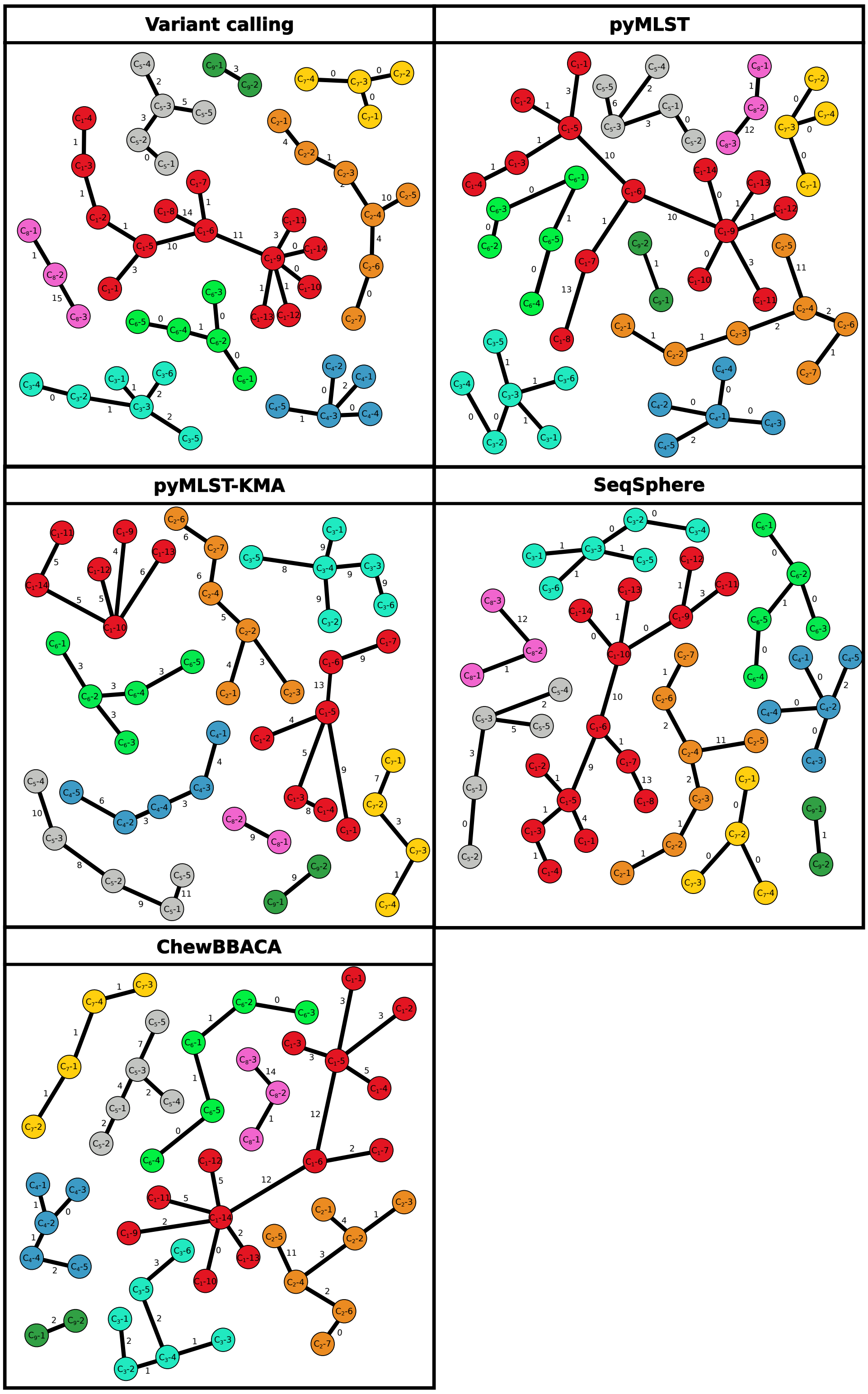

Fig. S5: Minimum spanning trees of *61 Pseudomonas aeruginosa* core genomes using pyMLST, SeqSphere, ChewBBACA, pyMLST-KMA and the variant calling approach. Only genes present in more than 95% of the genomes in our collection were retained.
